# Characteristics of Sudden Unexpected Infant Deaths — United States, 2020–2024

**DOI:** 10.15585/mmwr.mm7535a1

**Published:** 2026-09-10

**Authors:** Alexa B. Erck Lambert, Sharyn E. Parks, Carri Cottengim, Yousra A. Mohamoud, Kathleen N. Ly, Charlan D. Kroelinger, Tiffany Riehle-Colarusso

**Affiliations:** ^1^Division of Reproductive Health, National Center for Chronic Disease Prevention and Health Promotion, CDC; ^2^Partner Engagement and Data Dissemination Branch, Division of Vital Statistics, National Center for Health Statistics, CDC.

**Summary**

**What is already known about this topic?**

Circumstances-at-death data from the Sudden Unexpected Infant Death (SUID) and Sudden Death in the Young Case Registry (case registry) complement vital statistics and are critical to prevention of SUIDs.

**What is added by this report?**

Analysis of 2020–2024 case registry data found that SUID sociodemographic characteristics and risk factors were similar to those identified in previous reports: most deaths occurred in infants who were aged 0–3 months (66%), male (57%), Black or African American (38%) or White (37%), born at ≥37 weeks’ gestation (74%), and insured by Medicaid (69%). Unsafe sleep factors were identified in ≥73% of cases. Almost all SUIDs had an autopsy and death investigation; completeness varied.

**What are the implications for public health practice?**

Case registry data are critical to improving death investigations, etiologic and risk factor understanding, and evidence-based SUID prevention.

## Abstract

Sudden unexpected infant death (SUID) accounts for approximately 3,400 deaths among infants aged <1 year in the United States annually. To reduce SUID risk, the American Academy of Pediatrics recommends that infants be placed to sleep supine on a flat, firm, nonshared surface, without soft bedding. SUID vital statistics data do not include details on sleep environment and other contextual factors that are critical for SUID surveillance and prevention. CDC’s SUID and Sudden Death in the Young Case Registry (case registry) helps fill this gap. The case registry, a multijurisdictional, population-based active surveillance system, builds on child death review programs, compiling detailed information on deaths in persons aged 0–18 years from multiple data sources. CDC analyzed 2020–2024 case registry data to describe 1) SUID by sociodemographic and sleep environment characteristics, 2) the case registry classification algorithm SUID categories, and 3) death investigation components. Among the 4,861 SUIDs included in this analysis, most decedents were aged 0–3 months, male, Black or African American or White, born full-term, and insured by Medicaid. Unsafe sleep factors were present in at least three fourths of SUIDs. Almost all infants had an autopsy and death investigation, although completed components varied. This newly reported, detailed case registry information can be used by public health professionals and clinicians to improve death investigations, increase understanding of etiologies and risk factors, and enhance focused prevention efforts to reduce SUIDs.

## Introduction

Sudden unexpected infant death (SUID) includes sudden infant death syndrome (SIDS), accidental suffocation and strangulation in bed, and unknown causes of death. SUID accounts for approximately 3,400 deaths in infants (children aged <1 year) in the United States annually and disproportionately affects Black or African American (Black) and American Indian or Alaska Native infants. (Persons of Hispanic or Latino [Hispanic] origin might be of any race but are categorized as Hispanic; all racial groups are non-Hispanic.) To reduce SUID risk, the American Academy of Pediatrics (AAP) recommends that infants be placed to sleep supine on a flat, firm, nonshared sleep surface (i.e., crib or bassinet [crib]), without soft bedding (*1*). National vital statistics data demonstrate declines in SUID rates (number of SUIDs per 100,000 live births) during the 1990s, from 154.6 in 1990 to 93.9 in 1999. These declines were attributed to clinical guidance and public health campaigns about safe infant sleep practices (*1*); however, progress has stalled, and from 2020 to 2022, SUID rates increased from 92.9 to 100.9.

Vital statistics data are best for monitoring national SUID rates and exploring information available on birth and death certificates. However, they lack details about death circumstances and often do not contain sufficient information to determine SUID subtype (e.g., explained versus unexplained deaths) (*2*). CDC’s SUID and Sudden Death in the Young (SDY) Case Registry (case registry) aims to fill this gap. The case registry, which began with five jurisdictions in 2009, currently includes 32 jurisdictions and captures approximately two in five U.S. SUIDs. The case registry is a multijurisdictional, population-based, active surveillance system that compiles detailed information on sudden deaths in persons aged <20 years from multiple data sources, extending beyond publicly available information (e.g., vital statistics). The case registry builds on existing child death review programs* and uses the Health Resources and Services Administration and CDC-supported National Center for Fatality Review and Prevention's Pediatric National Fatality Review – Case Reporting System. A multidisciplinary child death review team reviews each SUID and develops case-specific prevention recommendations that are used with compiled data to guide SUID prevention locally and statewide. In addition, every SUID is categorized using a CDC-developed classification algorithm to differentiate explained from unexplained deaths, account for incomplete investigation information, and document whether unsafe sleep factors contributed to the death (*2*). This report describes 2020–2024 case registry SUID data, not previously published or available through vital statistics alone, building on previous evidence (*2*–*6*) to improve understanding and prevention of SUID.

## Methods

### Data Source

SUIDs^†^ that occurred during 2020–2024 among residents of 31 jurisdictions^§^ participating in the case registry and that met case registry data quality criteria (4,861) were included in this analysis.

### Analysis

A descriptive analysis was conducted. SAS (version 9.4; SAS Institute) was used to calculate frequencies and percentages of sociodemographic characteristics, sleep environment characteristics, classification categories (*2*), and investigation components. This activity was reviewed by CDC, deemed not research, and conducted consistent with applicable federal law and CDC policy.^¶^

## Results

### Characteristics of SUIDs

Among 4,861 case registry SUIDs that occurred during 2020–2024 and met case registry data quality criteria, most involved infants who were aged 0–3 months (66%), male (57%), Black (38%) or White (37%), born at full term (≥37 weeks’ gestation) (74%), and insured by Medicaid (69%) (Table 1). Infants’ sleep environment at death frequently involved unsafe sleep factors that were not mutually exclusive, including 46% of infants who were found in a nonsupine position, 75% on a sleep surface other than a crib, 78% with soft bedding, and 59% sharing a sleep surface. Full airway obstruction (nose and mouth, neck, or chest) was documented in 28% of SUIDs.

**TABLE 1:** Sudden unexpected infant deaths, by demographic characteristics of the infant and sleep environment characteristics — Sudden Unexpected Infant Death and Sudden Death in the Young Case Registry, United States, 2020–2024

Characteristic	No (%)
Demographic characteristics	4,861 (100)
Age at death, mos	
0–3	3,206 (66)
4–6	1,173 (24)
7–11	482 (10)
Sex*	
Male	2,762 (57)
Female	2,089 (43)
Missing^†^	10 (<1)
Race and ethnicity*^,§^	
American Indian or Alaska Native	86 (2)
Asian	39 (1)
Black or African American	1,828 (38)
Hispanic or Latino	650 (13)
Native Hawaiian or Pacific Islander	20 (<1)
White	1,820 (37)
Multiple races	260 (5)
Unknown/Missing^†^	158 (3)
Gestational age at birth, wks*	
<34	365 (8)
34–36	721 (15)
≥37	3,602 (74)
Unknown	124 (3)
Missing^†^	49 (1)
Health insurance at the time of death	
None	88 (2)
Medicaid^¶^	3,375 (69)
Private	703 (14)
Other/Combination**	229 (5)
Unknown	320 (7)
Missing^†^	146 (3)
**Sleep environment characteristics**	
Position in which the infant was found	
Supine	1,911 (39)
Prone	1,508 (31)
Side	715 (15)
Unknown	454 (9)
Missing^†^	273 (6)
Sleep location*	
Crib, portable crib, or bassinet	1,011 (21)
Adult bed or futon	2,756 (57)
Chair or couch	439 (9)
Other^††^	424 (9)
Unknown	75 (2)
Missing^†^	156 (3)
Soft bedding in sleep environment	
Yes	3,814 (78)
No	365 (8)
Unknown	383 (8)
Missing^†^	299 (6)
Surface sharing at time of death*^,§§^	
Yes	2,869 (59)
No	1,691 (35)
Unknown	124 (3)
Missing^†^	177 (4)
Infant airway when found*	
Unobstructed	1,463 (30)
Partially obstructed	464 (10)
Fully obstructed	1,347 (28)
Unknown	1,106 (23)
Missing^†^	481 (10)

* Percentages might not sum to 100 because of rounding.

^†^ A missing response indicates the variable was left blank during data entry, and no attempt was made to find the answer. An unknown response indicates the variable was considered; however, the information necessary to answer the question was not available. Guidance for Improving Child Death Review Data Quality

^§^ Race and ethnicity were missing for <1% of infants. Persons of Hispanic or Latino (Hispanic) origin might be of any race but are categorized as Hispanic; all racial groups are non-Hispanic.

^¶^ Although insurance categories are mutually exclusive, infants indicated as being insured by Medicaid might have also had additional insurance.

** Health insurance indicated as other, state plan, or Indian Health Service, or more than one was selected.

^††^ Other includes floor, car seat, stroller, bedside sleeper, inclined sleeper, swing, or bouncy chair.

^§§^ Surface sharing is defined as the infant sleeping with another person (i.e., infant, child, or adult) or animal on any surface (e.g., adult bed, crib, bassinet, or couch) at the time of death.

### Categorization of SUIDs

Using categories from the case registry classification algorithm (Box) (*2*), the analysis categorized SUIDs as follows: unexplained–no autopsy or death investigation (57; 1%), unexplained–incomplete case information (1,139; 23%), unexplained–no unsafe sleep factors (117; 2%), unexplained–unsafe sleep factors (no or unknown airway obstruction) (2,126; 44%), unexplained–possible suffocation with unsafe sleep factors (503; 10%), and explained–suffocation with unsafe sleep factors (919; 19%) (Table 2). Most SUIDs (3,548; 73%) were classified in a category indicating that unsafe sleep factors (*1*) were identified in the sleep environment; unsafe sleep factors might also have been present in the unexplained–no autopsy or death investigation and unexplained–incomplete case information categories. Among explained–suffocation, attributed (nonmutually exclusive) mechanisms included soft bedding (obstruction of the infant’s airway by soft objects or loose bedding in the immediate sleep environment) (681; 74%), overlay (obstruction of the infant’s airway by a person or animal) (223; 24%), wedging (obstruction of the infant’s airway resulting from being trapped between two inanimate objects) (69; 8%), or other (obstruction of the infant’s airway by something else in the sleep environment) (33; 4%).

BOX Sudden unexpected infant death categorization criteria* — Sudden Unexplained Infant Death and Sudden Death in the Young Case Registry, United States, 2020–2024

**Table:** 

**Sudden unexplained infant death category**	**Criteria that must be met**
**Unexplained**	
Unexplained–no autopsy or death scene investigation	Death scene investigation or autopsy not reported
Unexplained–incomplete case information	Location and position of the infant when found is unknown, or Toxicology, radiology, or pathology (i.e., histology, microbiology, or other pathology such as genetic testing) not performed during autopsy
Unexplained–no unsafe sleep factors	Death occurred while the infant was awake or during sleep If the infant was asleep, the infant was supine, in a crib or bassinet, on a nonshared, firm noninclined sleep surface, without any soft or loose bedding per American Academy of Pediatrics (AAP) Recommendations for Reducing Infant Deaths in the Sleep Environment
Unexplained–unsafe sleep factors	Death occurred in an unsafe sleep environment (per AAP recommendations); however, environmental contribution to death was uncertain because airway was unobstructed or unknown whether obstructed
Unexplained–possible suffocation with unsafe sleep factors	Death with unsafe sleep factors and documentation of at least a partial airway obstruction but missing one or more of the following criteria: 1. Nonconflicting, reliable witness account 2. No potentially fatal findings or concerning conditions^†^ 3. Strong evidence of a full, external airway obstruction 4. Suffocation probable given the infant’s age and stage of development
**Explained**	
Explained–suffocation with unsafe sleep factors	Death with unsafe sleep factors and meets all of the following criteria: 1. Nonconflicting, reliable witness account 2. No potentially fatal findings or concerning conditions^†^ 3. Strong evidence of a full, external airway obstruction 4. Suffocation probable given the infant’s age and stage of development

**Source**: Adapted from Shapiro-Mendoza CK, Camperlengo L, Ludvigsen R, et al. Classification system for the Sudden Unexpected Infant Death Case Registry and its application. Pediatrics 2014;134:e210–9. https://doi.org/10.1542/peds.2014-0180

* Categories are mutually exclusive and assigned in sequence using the Sudden Unexplained Infant Death and Sudden Death in the Young Case Registry classification algorithm; each category is applied only to cases remaining unclassified after the preceding criteria have been applied.

^†^ Potentially fatal findings or concerning conditions should be severe enough to independently cause death. However, in these cases, their contribution to the death is uncertain, creating doubt that the airway obstruction alone was the cause and raising the possibility that other factors (e.g., drug toxicity or severe respiratory illness) might have contributed.

**TABLE 2:** Sudden unexpected infant deaths, by mechanism of suffocation and characteristics of death investigation — Sudden Unexpected Infant Death and Sudden Death in the Young Case Registry, United States, 2020–2024

**SUID and SDY Case Registry classification category and mechanism of suffocation**	No. (%)*
**Total^†^**	**4,861 (100)**
**Explained, suffocation with unsafe sleep factors**	**919 (19)**
Soft bedding^§^	681 (74)
Overlay^¶^	223 (24)
Wedging**	69 (8)
Other^††^	33 (4)
**Unexplained**	**3,942 (80)**
No autopsy or death investigation	57 (1)
Incomplete case information	1,139 (23)
No unsafe sleep factors	117 (2)
Unsafe sleep factors	2,126 (44)
Possible suffocation with unsafe sleep factors	503 (10)
**Death investigation characteristics^†^**	
Autopsy performed	
Yes	4,779 (98)
No	53 (1)
Unknown	22 (<1)
Missing^§§^	7 (<1)
Death investigation performed^†^	
Yes	4,751 (98)
No	52 (1)
Unknown	51 (1)
Missing^§§^	7 (<1)
Death scene re-creation performed	
Yes, with doll	2,182 (45)
Yes, without doll	236 (5)
No	1,668 (34)
Unknown	452 (9)
Missing^§§^	323 (7)
Toxicology testing performed	
Yes	4,613 (95)
No	112 (2)
Unknown	89 (2)
Missing^§§^	47 (1)
Imaging performed^†^	
Yes	4,311 (89)
No	292 (6)
Unknown	134 (3)
Missing^§§^	124 (3)
Microbiology, histology, or pathology testing performed	
Yes	4,649 (96)
No	45 (1)
Unknown	114 (2)
Missing^§§^	53 (1)
Genetic testing performed	
Yes	681 (14)
No	3,215 (66)
Unknown	433 (9)
Missing^§§^	532 (11)

**Abbreviations: **SDY = Sudden Death in the Young; SUID = sudden unexpected infant death.

* Percentages are calculated using the total SUIDs as the denominator, except for the mechanisms of suffocation (i.e., soft bedding, overlay, wedging, or other), which are calculated from among the 919 SUIDs categorized as suffocation with unsafe sleep factors.

^†^ Percentages might not sum to 100 because of rounding.

^§^ Obstruction of an infant’s airway by blanket, sheet, pillow, couch or recliner cushions, or other soft objects or loose bedding that are part of the immediate sleep environment.

^¶^ Obstruction of an infant’s airway by a person or animal.

** Obstruction of an infant’s airway as a result of being stuck or trapped between two inanimate objects.

^††^ Obstruction of an infant’s airway by something in the sleep environment other than soft bedding, overlay, or wedging such as a plastic bag.

^§§^ A missing response indicates the variable was left blank during data entry, and no attempt was made to find the answer. An unknown response indicates the variable was considered; however, the information necessary to answer the question was not available. Guidance for Improving Child Death Review Data Quality

### SUID Death Investigation

Overall, among SUIDs in the case registry, 4,779 (98%) had an autopsy and 4,751 (98%) had a death investigation, including toxicology testing (4,613; 95%); imaging (4,311; 89%); microbiology, histology, or pathology testing (4,649; 96%); and death scene re-creation** with the person who found the infant unresponsive (2,418; 50% [90% of re-creations made use of a doll]) (Table 2). Genetic testing was not documented for 86% of infants. Information was missing or unknown regarding the position in which the infant was found for 15% of SUIDs and regarding airway obstruction status for 33% (Table 1).

## Discussion

Findings from this analysis of the most recent SUID case registry data are consistent with previously reported findings of sociodemographic and sleep environment characteristics, classification categories, and completed investigation components (*2*–*6*). Case registry data can offer insight into the drivers of persistent SUID rates that vital statistics data alone cannot. Continued use of recent case registry data is critical to improving death investigations, increasing understanding of SUID etiologies and risk factors, and providing contextual information for SUID prevention efforts.

Case registry data on the thoroughness of autopsies and death scene investigations, reflected in the unexplained–incomplete case information category (23%), can identify areas for improvement and support engagement with medicolegal communities. Consistent with previous case registry analyses (*3*), this analysis found that nearly all case registry SUIDs during 2020–2024 had an autopsy (98%) and scene investigation (98%), and critical autopsy components (e.g., toxicology, imaging, or pathology) were typically completed. However, completeness of critical contextual information varied, including information regarding the position in which the infant was found and presence or absence of airway obstruction. Approximately one half of investigations had a documented death scene re-creation, conducted by investigators, with the witness present, to capture circumstances at death. Use of a doll during re-creation to assist witnesses in demonstrating the exact position in which the infant was found increased from 37% during 2010–2012 to 45% in this analysis (*3*). Although room for improvement remains, this increase might reflect case registry efforts to improve death investigations.

Approximately one half of SUIDs were categorized as unexplained–unsafe sleep factors or unexplained–possible suffocation with unsafe sleep factors. More complete information on airway obstruction status would improve differentiation between explained sleep-related suffocation, with clear prevention implications, and unexplained death for which further investigation of etiologies and risk factors are needed.

The small but heterogeneous unexplained–no unsafe sleep factors category might reflect physiologic or genetic etiologies or those unrelated to the sleep environment (*4*). In addition, approximately one third of SUIDs had documentation of an unobstructed airway, again suggesting an etiology unrelated to suffocation. These findings underscore the ongoing need to investigate contributing factors that are unrelated to the sleep environment and expand diagnostic evaluation of SUIDs, including relevant genetic testing during autopsy.

Case registry data have previously indicated that unsafe sleep factors increase the risk for explained sleep-related suffocation and unexplained death (*5*). Most SUIDs in this analysis, regardless of whether they were explained or unexplained, had unsafe sleep factors present, and approximately one fifth were categorized as explained–suffocation. The ≥73% prevalence of unsafe sleep factors identified among SUIDs in this analysis was similar to that reported for 2011–2020 (≥76%) (*6*). Compared with live infants in the 2016 Pregnancy Risk Assessment Monitoring System (*7*), SUID cases less frequently met AAP’s safe sleep recommendations, reinforcing the importance of safe sleep practices to reduce SUID risk.

Although safe sleep recommendations^††^ are widely promoted, supporting caregivers in implementing recommended practices can be complex. SUIDs differ by race and ethnicity, and Medicaid coverage was overrepresented among SUIDs in the analysis compared with 2020–2024 births among case registry jurisdictions (69% versus 39%).^§§^ State or local case registry data analyses can identify prevalent risk factors, populations at high risk for SUID, and contextual contributors (e.g., parental unemployment or housing instability) to guide development and implementation of focused prevention efforts (*8*). CDC supports the development of such community-based, data-guided SUID prevention efforts in 10 case registry jurisdictions. These jurisdictions use local data, engage with families and providers in their communities, identify locally relevant barriers to and facilitators for practicing safe infant sleep, and develop tailored strategies to increase adoption of safe sleep practices (*9*).

### Limitations

The findings in this report are subject to at least four limitations. First, case registry data are subject to availability, accuracy, and biases in primary data sources, including caregiver and investigator accounts of often unwitnessed events and chaotic death scenes. This might result in higher percentages of missing and unknown responses to some critical variables. Second, social desirability bias might result in underreporting of unsafe sleep practices (*10*), which could lead to underrepresentation of unsafe sleep factors among SUIDs. Third, although reporting of most variables was >95% complete; variables with >5% missing or unknown responses warrant cautious interpretation, because incomplete information might affect validity and generalizability of reported data. Finally, the study population comprised SUIDs in 31 participating case registry jurisdictions which accounted for approximately 40% of U.S. SUIDs. Thus, study results are not fully generalizable limits generalizability to other jurisdictions and nationally; however, sociodemographic characteristics of the case registry population were similar to characteristics of all U.S. SUIDs.

### Implications for Public Health Practice

Public health professionals and clinicians can use newly reported case registry data to improve death investigations, increase understanding of etiology and risk factors, and enhance prevention efforts. Improving the completeness of death investigations, including performing genetic testing and documenting airway obstruction status, can better distinguish deaths with clear prevention implications. SUIDs with no unsafe sleep factors present at the time of death highlight the need to explore additional risk factors and underlying etiologies. Continued analysis and dissemination of up-to-date case registry data offer a more relevant reflection of current practice, which might more effectively guide SUID understanding and prevention.

## Footnotes

* The SUID and SDY Case Registry was built on existing child death review programs and protocols, and data are housed in the Pediatric National Fatality Review-Case Reporting System.

† SUIDs included deaths with any of the following causes reported on the death certificate: unknown; undetermined; SIDS; SUID; unintentional sleep-related asphyxia, suffocation, strangulation; unspecified suffocation; cardiac or respiratory arrest without other well-defined causes; or nonspecific causes (e.g., respiratory illness) with potentially contributing unsafe sleep factors. Intentional homicides are excluded.

§ Case registry jurisdictions with cases included in the analysis are Alaska; Arizona; Sacramento County, California; San Francisco County, California; Colorado; Delaware; DeKalb and Fulton counties, Georgia; Canyon County, Idaho; Cook County, Illinois; Indiana; Kansas; Kentucky; Louisiana; Maryland; Michigan; Minnesota; Nevada; New Hampshire; New Jersey; New Mexico; Hamilton County, Ohio; select counties in Pennsylvania; Philadelphia County, Pennsylvania; Rhode Island; select counties in South Carolina; South Dakota; Tennessee; Utah; select counties in Virginia; Pierce County, Washington; and Wisconsin.

¶ 45 C.F.R. part 46.102(l)(2), 21 C.F.R. part 56; 42 U.S.C. Sect. 241(d); 5 U.S.C. Sect. 552a; 44 U.S.C. Sect. 3501 et seq.

** Death scene re-creation, a key part of a complete investigation, is conducted with the person who found the infant unresponsive, to document the circumstances surrounding the death and assist the pathologist in determining the cause of death (SUID Investigation, Chapter 7).

†† The Safe to Sleep campaign, led by the National Institutes of Health National Institute of Child Health and Human Development, is an evidence-based national effort to reduce the risk for SUID.

§§ Births were restricted for each jurisdiction to the years in which that jurisdiction contributed case registry data. Counties were included when available in CDC WONDER nativity data. Data are current as of September 1, 2026.
